# A Novel Path Planning Algorithm for Truck Platooning Using V2V Communication

**DOI:** 10.3390/s20247022

**Published:** 2020-12-08

**Authors:** Yongki Lee, Taewon Ahn, Chanhwa Lee, Sangjun Kim, Kihong Park

**Affiliations:** 1 Graduate School of Automotive Engineering, Kookmin University, Seoul 02707, Korea; yklee0731@kookmin.ac.kr (Y.L.); atw6754@gmail.com (T.A.); 2 Research and Development Division, Hyundai Motor Company, Gyeonggi-do 18280, Korea; chanhwa.lee@gmail.com (C.L.); rixoest@hyundai.com (S.K.)

**Keywords:** TROOP, truck platooning, path planning, kalman filter, V2V communication, string stability, off-tracking, articulated cargo trucks, kabsch algorithm

## Abstract

In truck platooning, the leading vehicle is driven manually, and the following vehicles run by autonomous driving, with the short inter-vehicle distance between trucks. To successfully perform platooning in various situations, each truck must maintain dynamic stability, and furthermore, the whole system must maintain string stability. Due to the short front-view range, however, the following vehicles’ path planning capabilities become significantly impaired. In addition, in platooning with articulated cargo trucks, the off-tracking phenomenon occurring on a curved road makes it hard for the following vehicle to track the trajectory of the preceding truck. In addition, without knowledge of the global coordinate system, it is difficult to correlate the local coordinate systems that each truck relies on for sensing environment and dynamic signals. In this paper, in order to solve these problems, a path planning algorithm for platooning of articulated cargo trucks has been developed. Using the Kalman filter, V2V (Vehicle-to-Vehicle) communication, and a novel update-and-conversion method, each following vehicle can accurately compute the trajectory of the leading vehicle’s front part for using it as a target path. The path planning algorithm of this paper was validated by simulations on severe driving scenarios and by tests on an actual road. The results demonstrated that the algorithm could provide lateral string stability and robustness for truck platooning.

## 1. Introduction

**Figure 1:**
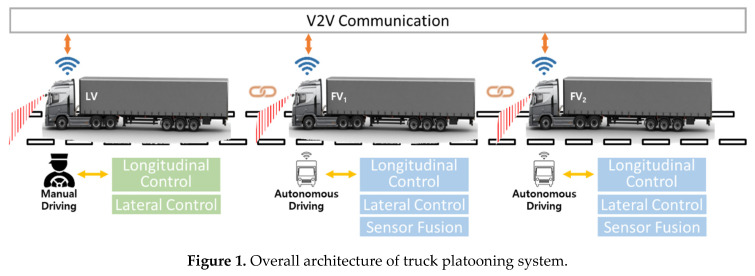
Overall architecture of truck platooning system.

Truck platooning refers to a form in which a number of trucks run as a fleet with short inter-vehicle distance using V2V (Vehicle-to-Vehicle) communication. Figure 1 shows the architecture of a truck platooning system. The leading vehicle (LV) is driven manually by an experienced driver, and the following vehicles (FVs) run by autonomous driving. The following vehicle (FV) uses environment sensors such as radar and camera to perceive vehicles and lanes ahead and perform autonomous driving by longitudinal and lateral vehicle control. The autonomous driving algorithm of the FV does not rely on GPS since the vehicle cannot receive correct GPS signals in some conditions, like when driving through tunnels. In the case of platooning of large cargo trucks, the length of the fleet can easily reach 100 m. Thus, the number of trucks in one platoon is usually limited to 3 or 4, considering the safety of the nearby vehicles.

Many studies are being conducted worldwide on truck platooning since it can bring improvement in driving safety, driver convenience, traffic throughput, fuel economy, and emission reduction. In Europe, truck manufacturers have been establishing consortiums for collaborative research on truck platooning. In 2016, they hosted the European Truck Platooning Challenge [1], and in 2018, they launched a large-scale inter-country project ENSEMBLE for multi-brand truck platooning [2]. In the US, the legislation necessary for platooning is actively being prepared to spur truck platooning to practical use in the near future [3].

In Korea, a first government project on truck platooning-TROOP (TRuck platOOning Project)-was launched in 2018 [4]. It will last until the end of 2021, with the final goal of developing the most advanced truck platooning system covering not just the control technologies but also the operational and management technologies based on the C-ITS services that the government has already established.

**Figure 2:**
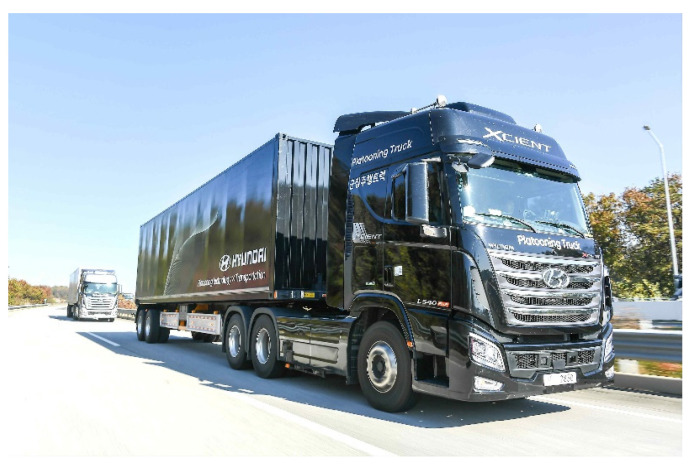
Photo of trucks in the TRuck platOOning Project (TROOP) Project. (Pictured by Hyundai Motor Company).

Figure 2 shows the photo of the trucks developed in the TROOP project. They are an articulated cargo truck with two bodies-tractor and trailer-linked by a kingpin where all the longitudinal and lateral controls are performed only at the tractor. The total length of each truck is 16.66 m. In the TROOP project, the ODD (Operational Design Domain) of truck platooning includes highway driving with a radius of 460R or more at a design speed of 90kph. This paper introduces the research on the path planning algorithm of the FV, which has been developed as part of the TROOP project.

The longitudinal control of the FV aims at maintaining a short distance to the front truck, and this is basically done by adopting the well-established ACC (Adaptive Cruise Control) algorithm, which relies on radar. Short inter-vehicle distance is important in truck platooning since it gives fuel economy to the FV by reducing aerodynamic drag. In TROOP, the final target is 0.5 s time-gap at 90 kph, or 12.5 m, which is shorter than the truck’s length. The FV also uses V2V communication, thus, it can immediately respond to critical situations such as when the LV makes sudden braking.

The lateral control of the FV aims at following the driving path of the LV while staying on its own lane. The FV uses a camera to perform lane keeping, but the well-established LKS (Lane Keeping System) algorithm [5,6] cannot be used since the front-view range of the camera is severely limited by the preceding truck [7]. The path following control of the FV sets as its target the trajectory that the LV has gone through. Therefore, in truck platooning, a higher level of control technology is required than the lateral control method used in general autonomous driving. There are two main methods for lateral control of platooning, ‘Direct vehicle-following’ and ‘Vehicle path-following’ [8].

In the direct vehicle-following method, the following vehicle directly follows the preceding vehicle by calculating the steering angle based on a geometrical principle using the relative longitudinal and lateral distance with the preceding vehicle [9,10]. Alternatively, using the relative position and relative angle between the subject vehicle CG (Center of Gravity) and the rear center of the preceding vehicle, a virtual curved path to the rear of the preceding vehicle can be computed [11,12]. However, since these methods use the relative position information of the rear of the preceding vehicle, not the trajectory of the steering wheel of the preceding vehicle, there may be a problem of driving inside the actual trajectory of the preceding vehicle during turning. In addition, when driving on a highway with a small curvature, since the relative yaw angle with the preceding vehicle is quite small, the reliability of the virtual curved path for following the preceding vehicle cannot be guaranteed if the accuracy of perception is low or the resolution of the measured value is small.

On the other hand, vehicle path-following is a method of following the trajectory of the preceding vehicle. The trajectory of the preceding vehicle can be obtained using motion parameters of the subject vehicle and storing the position coordinates of the rear of the preceding vehicle [8,13,14]. As the look-ahead distance within the trajectory of the preceding vehicle can be controlled, the performance of path tracking can be improved. However, there is a problem that enough look-ahead distance cannot be obtained at high speed due to a short inter-vehicle distance during platooning. In case of a semi-trailer truck, off-tracking, which is the difference in the path between the tractor’s steering axle and the trailer’s rear bumper, occurs during turning, causing a tracking error when following the preceding vehicle.

**Figure 3:**
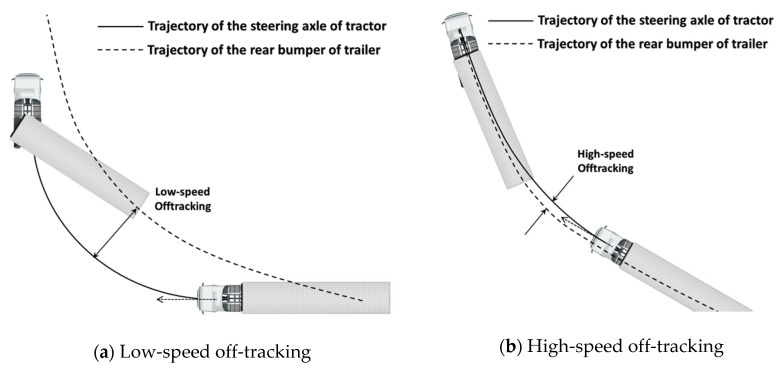
Schematic of off-tracking [15].

Figure 3 shows the off-tracking phenomenon, which occurs when a tractor-and-trailer type truck runs on a curved road. It displays different patterns at low speed and high speed for the same truck. At low speed, the trajectory of the rear bumper of the trailer is formed inside the trajectory of the tractor, while at high speed, the trajectory of the trailer travels outward than that of the tractor due to the increase in lateral acceleration [15]. Off-tracking is a major factor that harms the stability of the lateral dynamics of the platoon, and the stability gets worse as it propagates towards the tail of the platoon.

To overcome the off-tracking problem, the FV needs to use the trajectory of the tractor—not the trailer—of the preceding vehicle for its own target path [16]. However, it is not possible for the FV to perceive the position of the preceding vehicle’s tractor only by camera. A study has been proposed to take advantage of the curvature of the trajectory of the preceding vehicle’s trailer position [17], but it is effective only when the yaw angle between the two vehicles is small. Another study has been proposed to use the DRTK (Dynamic Based Real-Time Kinematic) and V2V to access the global position of the tractor of the preceding vehicle [18,19], but platooning trucks generally do not employ GPS since GPS signal cannot be received in conditions such as when driving through tunnels.

In this study, a path planning algorithm has been developed for lateral control of the FV in truck platooning formed by articulated cargo trucks. The algorithm uses camera/radar fusion data, IVN (In-Vehicle Network) chassis signals, and V2V communication. Using the Kalman filter and a novel coordinate conversion method, the FV is now able to figure out the trajectory of the LV’s tractor position to use as its own target path. The algorithm of this paper was validated by simulations on severe driving scenarios and by tests on an actual road. The results demonstrated that the algorithm can provide lateral string stability and robustness in truck platooning.

In addition, the proposed path planning algorithm can be expanded by generating a target path in the interchange and junction of the highway with a small turning radius and can be applied to various specially equipped vehicles as well as trucks. Furthermore, since the target path is generated based on the trajectory of the preceding vehicle using V2V communication, it can be applied even on an unpaved road without a lane. As a result, this study is expected to improve stability and fuel economy through platooning by applying it to various specially equipped vehicles in various road environments as well as large cargo trucks.

## 2. System Architecture

**Figure 4:**
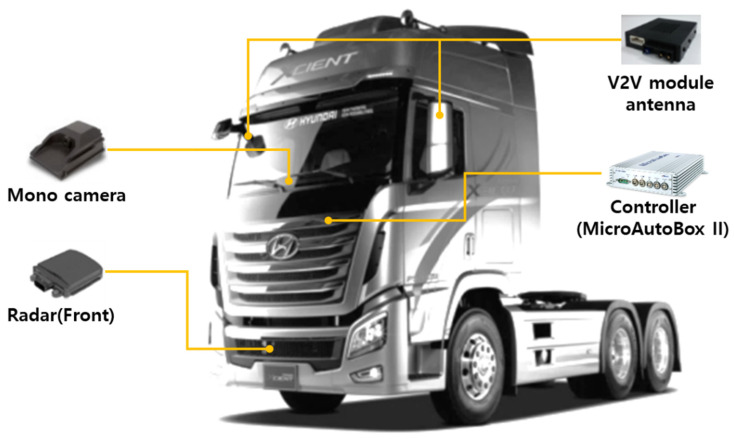
Hyundai Xcient 6 × 2 tractor.

Figure 4 shows Hyundai Xcient 6 × 2 tractor used in the TROOP project. The actuating mechanisms for steering, braking, and acceleration have been modified to enable autonomous driving. A mono camera and a radar are mounted on the dashboard and front bumper, respectively, to perceive the center point of the preceding vehicle’s bumper. The V2V module uses dual antennas, and they are installed inside the left and right side-mirrors to minimize the area of communication blind spot. Computation of the proposed path planning algorithm is carried out using MicroAutoBox II, which also serves as a CPU (Central Processing Unit) for implementing platooning control logics.

**Figure 5:**
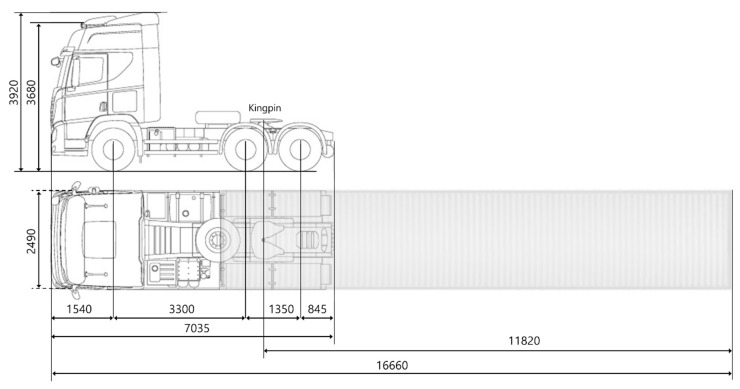
Specification of whole truck (unit: mm).

Figure 5 shows the specification of the truck used in the TROOP project. The total length of the vehicle is 16.66 m. The tractor and trailer are connected by a kingpin, but all the longitudinal and lateral controls are performed only at the tractor.

In the TROOP project, the truck platooning system was developed in three parts: Platooning operation control system, longitudinal control system, and lateral control system. The platooning operation control system performs the function of join, maintain, leave, and gap change of the platoon vehicle. In this paper, we only deal with the path planning method for lateral control, not the platooning operation system and the longitudinal control system.

**Figure 6:**
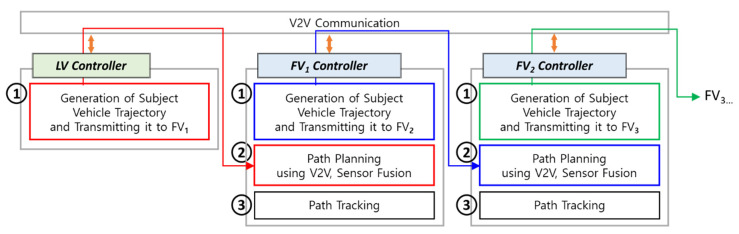
Overall architecture of truck platooning lateral controller.

Figure 6 shows the overall architecture of the truck platooning lateral controller. All vehicles participating in the fleet perform V2V communication among each other using DSRC/802.11p WAVE (Wireless Access in Vehicular Environment) protocol [20].

In Figure 6, the LV controller creates its driven trajectory (①) and transmits it to the FV via V2V communication. Using the LV’s trajectory, the FV performs path planning (②), i.e., calculates its own target path to follow. Finally, the FV implements path tracking control to follow the target path (③). In the same way, target paths are created between any adjacent FVs, so in essence, all FVs can follow the trajectory of the LV. Although the path tracking algorithm was developed as well in the TROOP project, this paper will cover only the path planning algorithm.

In order for the FV to create its own target path using the proposed path planning algorithm, longitudinal speed, lateral speed, yaw rate, and kingpin angle are required. Among them, vehicle speed, yaw rate, and kingpin angle can be measured, but their values are vulnerable to sensor noise. In addition, lateral speed cannot be directly measured. In order to solve this problem, the Kalman filter was designed in this study, and its details will be covered in Chapter 3.

Since all signals measured by each truck are measured in its own coordinate system, the driving trajectory of the preceding vehicle received via V2V communication must be converted to fit the local coordinate system of the recipient truck. This is also necessary since truck platooning in the TROOP project does not rely on any global positioning equipment. To solve this problem, a novel point matching method was developed in this study, and its details will be covered in Chapter 4.

If V2V communication is disconnected during platooning, platooning operation, and longitudinal/lateral control cannot be performed. The supervisor controller cancels platooning, and the control mode of each vehicle is changed to the independent autonomous driving mode, and the situation is notified to the driver. For example, the longitudinal control mode is changed to the ACC mode and lateral control mode to LKS. In this paper, we are dealing with the path planning method in the situation where V2V communication is operating normally.

## 3. Generation of Subject Vehicle Trajectory

**Figure 7:**
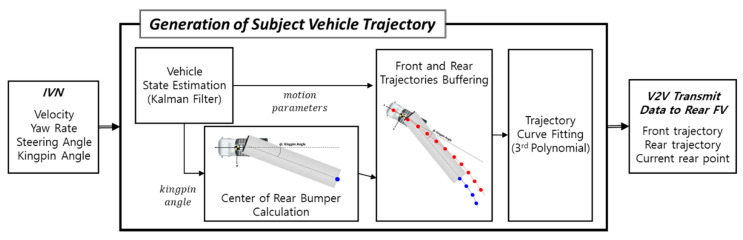
Architecture of subject vehicle trajectory generation.

Described in this chapter is how each truck creates its own driving trajectory, and the overall architecture is shown in Figure 7. Explanation in this chapter may be based on the LV, but the same method equally applies to all FVs.

### 3.1. Vehicle State Estimation by Kalman Filter

**Figure 8:**
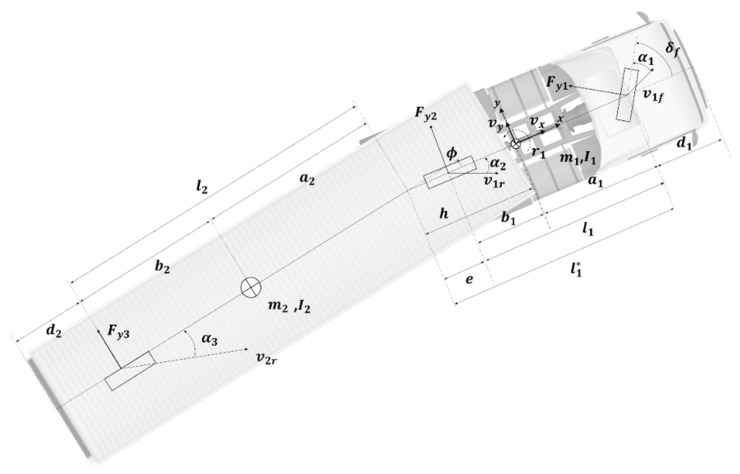
3 degrees of freedom articulated vehicle model.

This section explains the Kalman filter that was designed in this study to estimate the state variables needed to generate the subject vehicle trajectory. In previous studies, since it is impossible to measure the lateral speed of a vehicle, only general highway driving scenarios with a small lateral speed were considered, and the lateral speed was assumed to be zero [8,13,14]. However, the large cargo truck, which is the target vehicle of this study, has sensitive dynamic characteristics depending on the load weight and the road environment, and reliability of the buffered trajectory is important to consider not only driving within a lane but also a lane change scenario. Therefore, it is necessary to generate a trajectory more accurately in consideration of the lateral speed of the vehicle. There is a method of using a kinematic model of a vehicle [21], but as described in Chapter 1: Introduction, the kinematic model cannot represent the off-tracking characteristics of a truck. Thus, a 3 DOF (Degrees of Freedom) articulated vehicle model was selected to represent the dynamic characteristics of a truck properly. Figure 8 shows the 3 DOF articulated vehicle model from which the Kalman filter has been built. All variables and parameters of the model are defined with respect to the local coordinate system of each truck, which has its origin at the tractor CG, x-axis facing front, and *y*-axis facing left.

The articulated vehicle model in Figure 8 is represented by the state vector ${\left[v_{y1},\gamma_{1},\dot{\phi ,}\phi \right]}^{T}$. Here, $v_{y1}$ is the lateral speed of the tractor, $\gamma_{1}$ is the yaw rate of the tractor, and $\dot{\phi }$ and ϕ are the angular velocity and angle of the kingpin, respectively. Equation (1) shows the equations of motion of this articulated vehicle model.

(1) $$\begin{array}{l} (m_{1}+m_{2})({\dot{v}}_{y1}+v_{x}\gamma_{1})-m_{2}(h_{1}+a_{2}){\dot{\gamma }}_{1}-m_{2}a_{2}\ddot{\phi }\\ =-\frac{1}{v_{x}}[Cv_{y1}+\{C_{s_{1}}-C_{3}(h_{1}+a_{2}+b_{2}\}\gamma_{1}-C_{3}(a_{2}+b_{2})\dot{\phi }]+C_{1}\delta_{f}\\ -h_{1}m_{2}({\dot{v}}_{y1}+v_{x}\gamma_{1})+\{I_{1}+m_{2}h_{1}(h_{1}+a_{2})\}{\dot{\gamma }}_{1}+m_{2}h_{1}a_{2}\ddot{\phi }\\ =-\frac{1}{v_{x}}[C_{s_{1}}v_{y1}+\{C_{q_{1}^{2}}+C_{3}h_{1}(h_{1}+a_{2}+b_{2}\}\gamma_{1}+C_{3}h_{1}(a_{2}+b_{2})\dot{\phi }]+C_{1}a_{1}\delta_{f}\\ -m_{2}a_{2}({\dot{v}}_{y1}+v_{X}\gamma_{1})+\{I_{2}+m_{2}a_{2}(h_{1}+a_{2})\}{\dot{\gamma }}_{1}+(I_{2}+m_{2}a_{2}^{2})\ddot{\phi }\\ =-\frac{1}{v_{x}}[-C_{3}(a_{2}+b_{2})v_{y1}+\{C_{3}(a_{2}+b_{2})(h_{1}+a_{2}+b_{2})\}\gamma_{1}+C_{3}{\left(a_{2}+b_{2}\right)}^{2}(\dot{\phi }+v_{x}\phi )] \end{array}$$

In Equation (1), the subscripts ${\left(\right)}_{1}$ and ${\left(\right)}_{2}$ are used to denote the tractor and the trailer, respectively; m is the vehicle mass; I is the yaw moment of inertia; l is the wheelbase; a is the distance from CG to the front axle and b the distance from CG to the rear axle; h is the distance between the tractor’s CG and the kingpin; e is the distance between the tractor’s rear axle and the kingpin; and $l^{*}$ is the distance between the tractor’s front axle and the kingpin; $v_{x}$ is the longitudinal vehicle speed; $v_{y}$ is the lateral vehicle speed; and γ is the yaw rate. $C_{i}$, $\alpha_{i}$ and $F_{y_{i}}$ are the tire cornering stiffness, the wheel slip angle, and the lateral force of the $i^{th}$ wheel axle, respectively; and $\delta_{f}$ is the front steer angle.

Equation (1) can be made into a matrix-type state equation as below.

(2) $$\dot{X}=M^{-1}A\;X+M^{-1}B\;u\;where\;X={\left[v_{y1},\gamma_{1},\dot{\phi },\phi \right]}^{T},u=\delta_{f}$$

In the above, X is the state and u is the input, which is the front steer angle of the tractor. The parameter matrices in Equation (2) are defined as below.

(3) $$M=\;\left[\begin{array}{cccc} m_{1}+m_{2} & -m_{2}\left(h_{1}+a_{2}\right) & -m_{2}a_{2} & 0\\ -m_{2}h_{1} & I_{1}+m_{2}h_{1}\left(h_{1}+a_{2}\right) & m_{2}h_{1}a_{2} & 0\\ -m_{2}a_{2} & I_{2}+m_{2}a_{2}(h_{1}+a_{2}) & I_{2}+m_{2}a_{2}^{2} & 0\\ 0 & 0 & 0 & 1 \end{array}\right]\;A\;=\;-\frac{1}{v_{x}}\left[\begin{array}{cccc} C+C_{3} & C_{S_{1}}-C_{3}(h_{1}\;+\;l_{2})\;+\;(m_{1}\;+\;m_{2})v_{x}^{2} & -C_{3}l_{2} & -C_{3}v_{x}\\ C_{S_{1}}-C_{3}h_{1} & C_{q_{1}^{2}}+C_{3}h_{1}(h_{1}\;+\;l_{2})-m_{2}h_{1}v_{x}^{2} & C_{3}h_{1}l_{2} & C_{3}h_{1}v_{x}\\ -C_{3}l_{2} & C_{3}l_{2}(h_{1}\;+\;l_{2})-m_{2}a_{2}v_{x}^{2} & C_{3}l_{2}^{2} & C_{3}l_{2}v_{x}\\ 0 & 0 & -v_{x} & 0 \end{array}\right]\;B=\left[\begin{array}{c} \begin{array}{c} C_{1}\\ a_{1}C_{1} \end{array}\\ \begin{array}{c} 0\\ 0 \end{array} \end{array}\right]\;where\;C=C_{1}+C_{2},\;C_{s1}=a_{1}C_{1}-b_{1}C_{2},\;C_{q_{1}^{2}}=a_{1}^{2}C_{1}+b_{1}^{2}C_{2}$$

The Kalman filter of this study has been designed to estimate the state every 10 ms, which is equal to the CAN communication period in each vehicle. To do this, Equation (2) was converted into its discrete-time form as in Equation (4).

(4) $$X_{k+1}=A_{d}\cdot X_{k}+B_{d}\cdot \delta_{f}\;where$$

$$A_{d}=\left(I+\frac{\Delta t}{2}M^{-1}A\right){\left(I-\frac{\Delta t}{2}M^{-1}A\right)}^{-1},\;B_{d}=\Delta t\cdot M^{-1}B\;with\;\Delta t=0.01$$

In this study, the yaw rate and the kingpin angle were measured and used as input to the Kalman filter. Equation (5) shows the measurement model for the Kalman filter where $z_{k}$ is the measurement variable.

(5) $$z_{k}=H\cdot X_{k}\;where\;H=\left[\begin{array}{cc} \begin{array}{cc} 0 & 1\\ 0 & 0 \end{array} & \begin{array}{cc} 0 & 0\\ 0 & 1 \end{array} \end{array}\right]$$

The Kalman filter operates by repeating a series of two stages: Prediction and update [22]. Using the system model, it predicts the state variables, compensates for the difference between the measured variables and their predicted values, and outputs a new estimation of the state variables

In the prediction stage, the predicted state estimate ${\hat{x}}_{k}^{-}$ is computed together with the predicted error covariance $P_{k}^{-}$ by the following equation.

(6) $${\hat{x}}_{k}^{-}=A_{d}{\hat{x}}_{k-1}+B_{d}u_{k-1}\;P_{k}^{-}=A_{d}P_{k-1}^{+}A_{d}^{T}+Q$$

In Equation (6), the overstrike ^ means an estimate of the corresponding variable, and the superscripts ${\left(\right)}^{-}$ and ${\left(\right)}^{+}$ denote the predicted estimate and updated estimate, respectively. Q is a diagonal matrix that represents the covariance of the process noise. Q is used as a tuning parameter with the influence that: A larger value for a certain diagonal element of Q makes estimation of the corresponding state variable more affected by the measurement variables. In this study, Q was chosen as Equation (7) for the reason that the lateral velocity $v_{y1}$, which is not directly measurable, should depend more heavily on the measured variables for its estimation than the remaining state variables do.

(7) $$Q=\left[\begin{array}{cccc} 45 & 0 & 0 & 0\\ 0 & 1 & 0 & 0\\ 0 & 0 & 1 & 0\\ 0 & 0 & 0 & 1 \end{array}\right]$$

In the update stage, the algorithm computes the measurement residual ${\tilde{y}}_{k}$ and the Kalman gain $K_{k}$ using Equation (8). The measurement residual is the difference between the actual measurement $z_{k}$ and its estimate $H{\hat{x}}_{k}^{-}$, and the Kalman gain is a weight matrix for updating the prediction in Equation (6).

(8) $${\tilde{y}}_{k}=z_{k}-H{\hat{x}}_{k}^{-}\;K_{k}=P_{k}^{-}H^{T}{\left(HP_{k}^{-}H^{T}+R\right)}^{-1}$$

In Equation (8), R is a 2 × 2 diagonal matrix representing the covariance of the measurement noise. A larger diagonal element implies a higher reliability of the corresponding measurement signal. In this study, R was chosen as Equation (9), reflecting the characteristics of the sensors used in the trucks of the TROOP project. The yaw rate sensor gave fairly precise measurement, while the kingpin angle sensor had notable hysteresis property and not very high resolution.

(9) $$R=\left[\begin{array}{cc} 1 & 0\\ 0 & 1.5 \end{array}\right]$$

Finally, the state estimate is updated to ${\hat{x}}_{k}^{+}$ using Equation (10), and at the next time step, it is used as ${\hat{x}}_{k-1}$ in Equation (6). Likewise, the error covariance matrix is updated to $P_{k}^{+}$, and it is used as $P_{k-1}^{+}$ at the next time step.

(10) $${\hat{x}}_{k}^{+}={\hat{x}}_{k}^{-}+K_{k}\tilde{y}\;P_{k}^{+}=\left(I-K_{k}H\right)P_{k}^{-}$$

**Figure 9:**
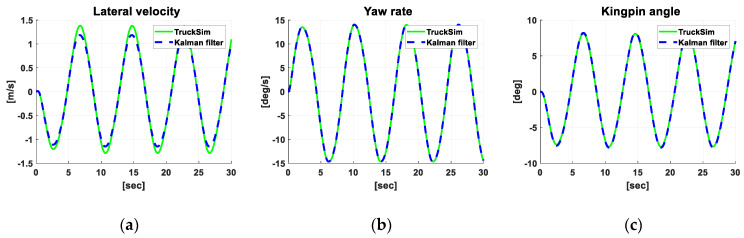
Comparison of simulation results of TruckSim and Kalman filter. (**a**) Lateral velocity; (**b**) Yaw rate; (**c**) Kingpin angle.

Figure 9 shows the results of the simulation, which was conducted to verify the performance of the Kalman filter. TruckSim was used for the vehicle model [23], and Matlab/Simulink was used to implement the filter algorithm. As input, a sinusoidal front steer angle with 90 deg amplitude and 0.125Hz frequency was applied to the truck model running at 90 kph.

Figure 9 shows that in the graph of lateral velocity, the estimation deviates from the true value (TruckSim) by as much as 12.6% in magnitude, but more importantly, there was little phase delay between the estimation and the true value. In the case of the yaw rate and kingpin angle, Figure 9 shows that their estimations are very close to their true values both in magnitude and phase.

Although there are more simulation results of the designed Kalman filter, they are not shown here since they all show similar credibility as above. The robustness of the Kalman filter was not actively examined in the simulation environment since the path planning test results with actual vehicles will demonstrate those properties anyway. The state estimation by the Kalman filter in this section is used for the path planning in Section 4.2 as well as for the subject vehicle trajectory generation in the following section.

### 3.2. Generation of Front and Rear Trajectories of Subject Vehicle

In the proposed path planning algorithm, the LV must generate its own driving trajectory since this trajectory makes it possible for the FV to create its target paths. This trajectory is composed of two parts - front trajectory $T_{F.LV}$ and rear trajectory $T_{R.LV}$-each of which is formed by accumulating into a buffer a total of 300 samples as below.

(11) $$T_{F.LV}=\left[\begin{array}{c} X_{F.LV}\\ Y_{F.LV} \end{array}\right]=\left[\begin{array}{c} x_{F.LV_{1}}\;x_{F.LV_{2}}\cdots \;x_{F.LV_{n}}\\ y_{F.LV_{1}}\;y_{F.LV_{2}}\cdots \;y_{F.LV_{n}} \end{array}\right]2\times 300\;\mathrm{matrix}\;T_{R.LV}=\left[\begin{array}{c} X_{R.LV}\\ Y_{R.LV} \end{array}\right]=\left[\begin{array}{c} x_{R.LV_{1}}\;x_{R.LV_{2}}\cdots \;x_{R.LV_{n}}\\ y_{R.LV_{1}}\;y_{R.LV_{2}}\cdots \;y_{R.LV_{n}} \end{array}\right]2\times 300\;\mathrm{matrix}$$

Throughout this paper, the subscript “*F*” (short for “Front”) refers to the center of the steering axle of the LV tractor, and the subscript “*R*” (short for “Rear”) refers to the center of the rear bumper of the LV trailer. In addition, the second subscript “*LV*” in ${\left(\;\right)}_{.LV\;}$ refers to the coordinate system in which the value is defined. For example, $x_{R.LV}$ means x values of “*R*” (= LV’s rear point) in terms of the LV local coordinate, $x_{R.FV}$ (which will be introduced later) means x values of “*R*” (= LV’s rear point) in terms of the FV local coordinate. Care must be taken not to be confused with the absolute concept of the first subscripts *F* and *R* and the relative concept of the second subscripts *LV* and *FV*, both used in the same variable.

**Figure 10:**
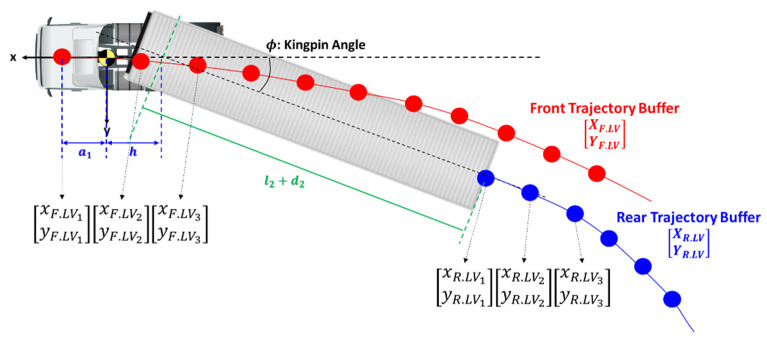
Subject vehicle trajectory buffer.

The numbering among 300 samples was made thus that the number increases from the most recent one to the past. The buffer is a pipeline with FIFO (First In First Out) property. Thus, ${\left(\;\right)}_{1}$ is the value at the current sample and ${\left(\;\right)}_{2}$ is the value at one sample before. When time passes to the next sample, all the elements of the buffer are shifted by one to the past, with the new sample added at the front. Figure 10 shows how the trajectory looks like from the view point of the truck.

Equation (12) shows the geometric equations of the “Front” and “Rear” points with respect to the LV’s local coordinate system. Since the local coordinate system on a truck changes as the truck moves, the trajectories $T_{F.LV}$ and $T_{R.LV}$ are updated at every sample with the information of translation and rotation made during the last sampling period.

(12) $$\left[\begin{array}{c} x_{F.LV_{1}}\\ y_{F.LV_{1}} \end{array}\right]=\left[\begin{array}{c} a_{1}\\ 0 \end{array}\right]\;\left[\begin{array}{c} x_{R.LV_{1}}\\ y_{R.LV_{1}} \end{array}\right]=\left[\begin{array}{c} -h\\ 0 \end{array}\right]\;+\;\left[\begin{array}{cc} \cos\phi & -\sin\phi \\ \sin\phi & \;\cos\phi \end{array}\right]\cdot \left[\begin{array}{c} -l_{2}-d_{2}\\ 0 \end{array}\right]$$

As seen above, the subject vehicle trajectory is represented by a total of 1200 points (counting front and rear trajectories and *x*-*y* values for each sample), which will be sent to the rear vehicles. The TROOP project mandates V2V communication to occur every 20 ms, but 1200 data points are too big for this purpose. To solve this problem, curve fitting to a third-order polynomial was performed, which reduces 600 data points to 4 coefficient values. Curve fitting also gives the side benefit of making the trajectory smooth even in the presence of outliers or random noises in the raw data of $T_{F.LV}$ and $T_{R.LV}$.

Table 1 shows the message that the LV transmits to the FV via V2V communication. Along with $T_{F.LV}$ and $T_{R.LV}$ in the form of the coefficients for their 3rd order polynomial, the LV sends to the FV [$x_{R.LV_{1}},\;y_{R.LV_{1}}]$, which is the center point of the LV’s rear bumper at the current sample. The is because the FV requires a reference point when implementing conversion between the local coordinates, which is explained in Chapter 4.

## 4. Proposed Path Planning Algorithm

**Figure 11:**
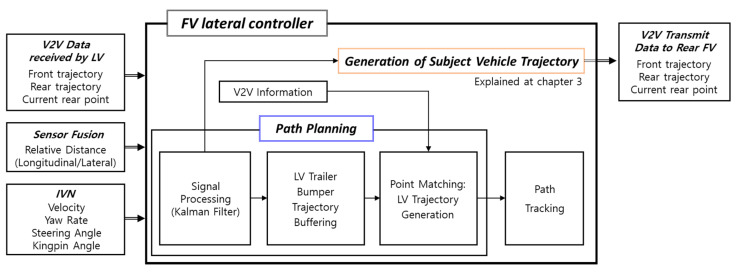
The architecture of the FV lateral controller.

Figure 11 shows the architecture of the FV lateral controller. Using the received message in Table 1, the FV performs path planning, i.e., calculates its target path, and this enters the path tracking control module as input.

The target path of the FV is basically the past trajectory of the LV, $T_{F.LV}$, but since $T_{F.LV}$ is defined from the viewpoint of the LV, it must be converted into the local coordinate system of the FV. This conversion is difficult without having any knowledge of the global coordinate system, or perhaps the most challenging part in the whole truck platooning. This chapter describes how this problem was solved in this research.

### 4.1. Concept of Coordinate Matching

**Figure 12:**
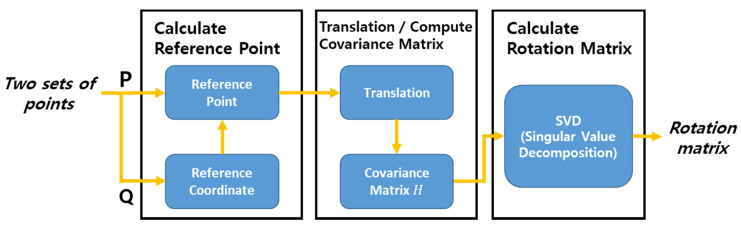
Workflow of point matching algorithm.

Figure 12 illustrates how the coordinate matching algorithm in this paper works, with an example of representing a dataset P in terms of the coordinate system of a dataset Q, when the two datasets P and Q have the same shape but are dislocated in the 2D plane.

Coordinate matching is equivalent to point matching among two datasets P and Q. First, a reference point is selected from P and Q, respectively. They must represent an identical point in an identical 2D shape. From the difference of their locations, a translation vector can be found, and all points in Q are translated accordingly. Finally, a rotation matrix is found that makes P and Q coincide.

**Figure 13:**
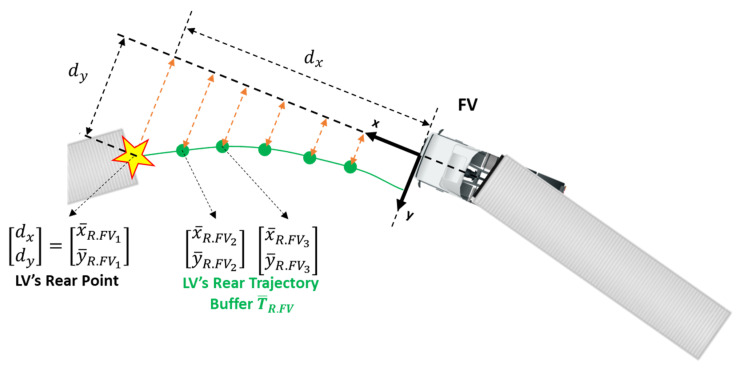
LV’s rear bumper trajectory from the viewpoint of FV.

To apply the above concept to path planning in the truck platooning, $T_{R.LV}$ and ${\bar{T}}_{R.FV}$ were chosen as P and Q in Figure 12, respectively. As previously explained, $T_{R.LV}$ is the trajectory of the LV’s rear point in the LV’s coordinate system. ${\bar{T}}_{R.FV}$, which appears for the first time here, represents the trajectory of the LV’s rear point in the FV’s coordinate system. As Figure 13 shows, the FV can generate ${\bar{T}}_{R.FV}$ since the FV can perceive the rear end of the LV with a camera and radar.

**Figure 14:**
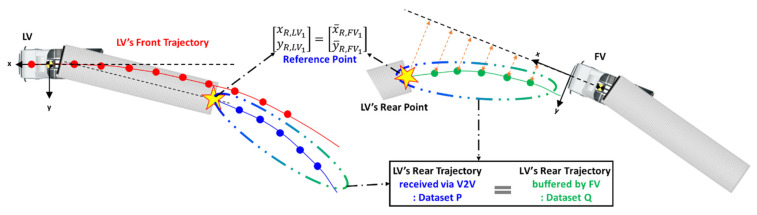
Schematics of coordinate matching in path planning of truck platooning.

For the reference point in the two datasets $T_{R.LV}$ and ${\bar{T}}_{R.FV}$, their first elements ${\left[x_{R.LV_{1}},\;\;y_{R.LV_{1}}\right]}^{T}$ and ${\left[{\bar{x}}_{R.FV_{1}},\;\;{\bar{y}}_{R.FV_{1}}\right]}^{T}$ were used since they represent an identical point at an identical time. Figure 14 shows the schematics of the coordinate matching between LV and FV.

### 4.2. Steps for Path Planning

This section shows how the FV computes its target path. Although the explanation is based on the LV–FV relationship, the same argument equally applies to any two adjacent trucks in the platoon, like in the ${\mathrm{FV}}_{1}$-${\mathrm{FV}}_{2}$ relationship. First, Table 2 shows four trajectories involved in path planning. It is reminded that the “Front” point refers to the center of the steering axle of the LV tractor, and the “Rear” point refers to the center of the rear bumper of the LV trailer.

**Figure 15:**
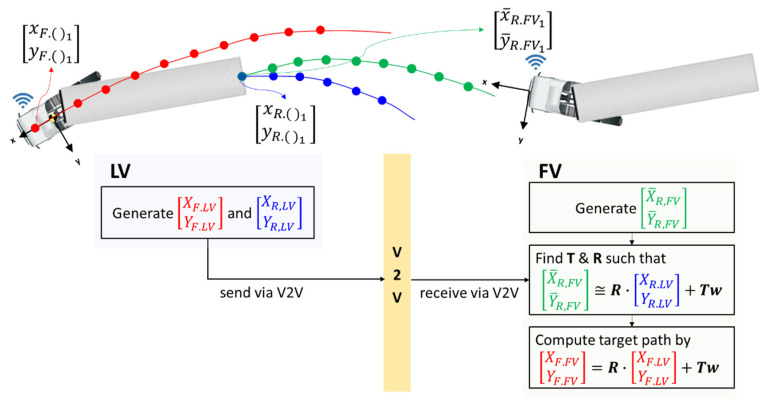
Schematics of path planning.

Figure 15 illustrates the process of how the FV performs path planning. First, the LV generates $T_{F.LV}$ and $T_{R.LV}$ and concurrently, the FV generates ${\bar{T}}_{R.FV}$. Through V2V communication, the FV receives $T_{F.LV}$ and $T_{R.LV}$ (in the form of the 3rd order polynomial coefficients) from the LV. Using $T_{R.LV}$ and ${\bar{T}}_{R.FV}$, the FV performs a coordinate conversion, which is possible since they represent an identical trajectory. Coordinate conversion is to find the translational and rotational relationship between LV and FV as in Equation (13).

Find 2×1 translation vector $T=\left[\begin{array}{c} x_{T}\\ y_{T} \end{array}\right]$ and 2×2 rotation matrix R such that

(13) $${\bar{T}}_{R.FV}≅R\cdot T_{R.LV}+T\cdot w\;where\;w=1\times n\;\mathrm{vector}\;\mathrm{with}\;\mathrm{ones}$$

Using the same relationship between the coordinate systems of LV and FV in the above, the FV can compute $T_{F.FV}$ by Equation (14).

(14) $$T_{F.FV}=R\cdot T_{F.LV}+T\cdot w$$

**Figure 16:**
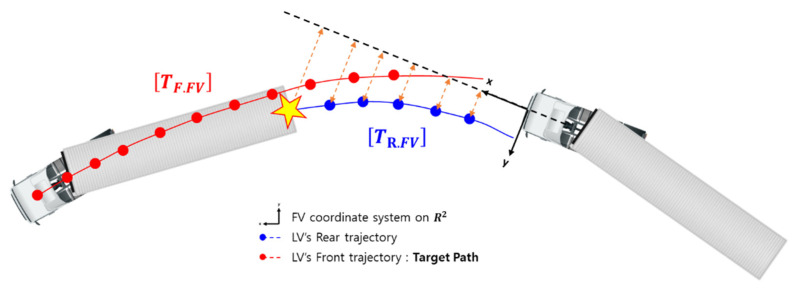
LV trajectory defined in the FV coordinate system.

$T_{F.FV}$ is the trajectory of the center of the steering axle of the LV’s tractor from the viewpoint of the FV, as seen in Figure 16. $T_{F.FV}$ is important because it can serve as the target path to the FV. However, the FV cannot generate $T_{F.FV}$ using only camera and radar since the tractor of the LV is blocked by the trailer of the LV most of the time. $T_{F.FV}$ is the final output of the path planning algorithm of this research.

### 4.3. Kabsch Algorithm

**Figure 17:**
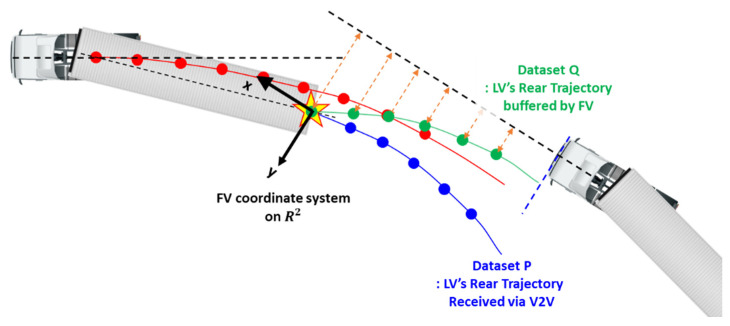
Reference point matching before finding the rotational relationship.

The rotation matrix in Equation (13) is computed after $T_{R.LV}$ is translated thus that its first point coincides with the first point of ${\bar{T}}_{R.FV}$, and this is illustrated in Figure 17.

In the study, the Kabsch algorithm [24,25] was adopted to find the optimal rotation matrix after the reference point matching. The Kabsch algorithm is a method to compute the optimal rotation matrix by minimizing the RMSD (Root Mean Squared Deviation) between the two datasets to be matched. This algorithm is used to convert the LV’s trajectory received by V2V communication into the FV’s coordinate system, and it is explained below in the context of path planning.

First, Equation (15) shows the two datasets and their reference points.

(15) $$P=T_{R.LV}=\left[\begin{array}{c} X_{R,LV}\\ Y_{R,LV} \end{array}\right]=\left[\begin{array}{c} x_{R.LV_{1}}\;x_{R.LV_{2}}\;\cdots \;x_{R.LV_{n}}\\ y_{R.LV_{1}}\;y_{R.LV_{2}}\;\cdots \;y_{R.LV_{n}} \end{array}\right],\;p_{0}=\left[\begin{array}{c} x_{R.LV_{1}}\\ y_{R.LV_{1}} \end{array}\right]:\;\mathrm{reference point of}\;P\;Q={\bar{T}}_{R.FV}=\left[\begin{array}{c} {\bar{X}}_{R,FV}\\ {\bar{Y}}_{R,FV} \end{array}\right]=\left[\begin{array}{c} {\bar{x}}_{R.FV_{1}}\;{\bar{x}}_{R.FV_{2}}\;\cdots \;{\bar{x}}_{R.FV_{n}}\\ {\bar{y}}_{R.FV_{1}}\;{\bar{y}}_{R.FV_{2}}\;\cdots \;{\bar{y}}_{R.FV_{n}} \end{array}\right],\;q_{0}=\left[\begin{array}{c} {\bar{x}}_{R.FV_{1}}\\ {\bar{y}}_{R.FV_{1}} \end{array}\right]:\;\mathrm{reference point of}\;Q$$

Next, the reference point matching in Figure 17 is done by translating both trajectories, thus that their reference points are located at the origin of the FV’s local coordinate system.

(16) $$\bar{P}=P-p_{0}\cdot w\;\bar{Q}=Q-q_{0}\cdot w$$

Next, the rotation matrix is computed thus that the root mean squared error between the two datasets $\bar{P}$ and $\bar{Q}$ are minimized. To do this, their covariance matrix H is formed and singular value decomposition is done to this matrix (Equation (17)).

(17) $$H=\bar{P}\cdot {\bar{Q}}^{T}\;H=U\cdot S\cdot V^{T}$$

Finally, the rotation matrix and the translation matrix T are computed by Equation (18), and using them the target path for the FV can be computed by Equation (14).

(18) $$d=sign\left(\det\left(V\cdot U^{T}\right)\right)\;R=V\cdot \left(\begin{array}{cc} 1 & 0\\ 0 & d \end{array}\right)\cdot U^{T}\;T=q_{0}-R\cdot p_{0}$$

Algorithm 1 shows the proposed path planning process.
**Algorithm 1** Path planning algorithm **Input:** $T_{F.LV}$: Trajectory of ”Front” point in LV’s coordinate system  $T_{R.LV}$: Trajectory of ”Rear” point in LV’s coordinate system, Dataset P  $T_{R.FV}$: Trajectory of ”Rear” point in FV’s coordinate system, Dataset Q  $\left[x_{R.LV_{1}},\;\;y_{R.LV_{1}}\right]$: Coordinate of LV’s “Rear” point at current sample **Output:** $T_{F.FV}$: Trajectory of ”Front” point in FV’s coordinate system *Find the reference points*
$p_{0}$*,*
$q_{0}$
*using*
$\left[x_{R.LV_{1}},\;\;y_{R.LV_{1}}\right]$ *Translate the trajectories to coincide with the origin of FV’s local coordinate system* $\bar{P}=P-p_{0}\cdot w$*,*
$\bar{Q}=Q-q_{0}\cdot w$*,*
w: *weight matrix* *Find the rotation matrix and translation matrix*
 $H=\bar{P}\cdot {\bar{Q}}^{T}$
← covariance matrix  [U, S, V]=svd(H)
← singular value decomposition  D
← 2-by-2 diagonal matrix  **if**
$\left|V\;\cdot U^{T}\right|<0$*,*
**then**
 D(2,2)=−1*,* return D; **end**
 $R=V\cdot D\cdot U^{T}$
←
*rotation matrix*
 $T=q_{0}-R\cdot p_{0}$
←
*translation matrix* *Compute the target path*
 $T_{F.FV}\leftarrow \;T_{F.LV}$
*using*
R
*and*
T **return**
$T_{F.FV}$;

## 5. Results of Simulation and Road Test Experiments

The proposed path planning algorithm of this paper was validated by both simulation and road test experiments, and their results are shown and analyzed in this chapter.

### 5.1. Simulation Result

A simulation environment was constructed to validate the path planning algorithm of this paper. The plant model for truck platooning was made with TruckSim, and the path planning logic was implemented with Matlab/Simulink. Table 3 shows three test scenarios used in the simulation. Scenarios S1 and S2 are the cases of driving on a curved road at low and high speeds, and Scenario S3 is a double lane change.

#### 5.1.1. Scenario S1–Curved Road with 100R, 40 kph, 0.7 s Time Gap

**Figure 18:**
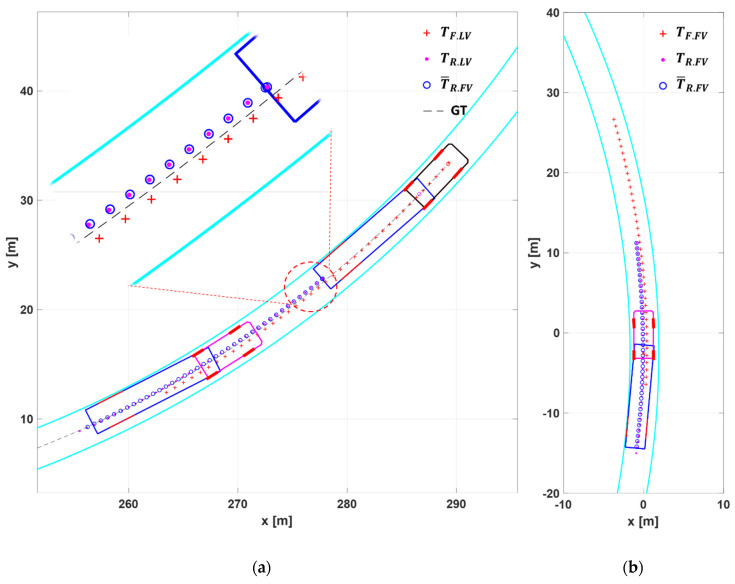
The simulation results for Scenario S1 (**a**) $T_{F.LV}$, $T_{R.LV}$, and ${\bar{T}}_{R.FV}$ in LV and FV; (**b**) $T_{F.FV}$, $T_{R.FV}$ and ${\bar{T}}_{R.FV}$ in FV. The black dash is the actual travel path (GT) of the front trajectory of LV. The cyan line next to the vehicles means the left/right lane.

Figure 18 shows the simulation results for Scenario S1.

Figure 18a shows that in the LV, the tractor trajectory $T_{F.LV}$ is formed inside the trailer trajectory $T_{R.LV}$, which verifies the pattern of the low-speed off-tracking illustrated in Figure 3a. An almost exact agreement of $T_{R.LV}$ and ${\bar{T}}_{R.FV}$ in Figure 18a indicates that the point matching algorithm using the two trajectories for the LV’s rear point was working successfully.

Comparing $T_{F.LV}$ and the trajectory of its true value (GT), the graph shows that they started at the same point, but $T_{F.LV}$ gradually deviates from GT as the point goes afterward. This is due to the estimation error in the Kalman filter, which was used in the generation of the subject vehicle trajectory. This kind of error cannot be completely avoided in any case. However, since the FV’s actual target path begins at some distance ahead—typically 16 m ahead at 40 kph—to secure enough look-ahead distance, this estimation error does not cause any significant trouble in path planning. In addition, this trajectory error can be reduced by giving more weight to the latest trajectory point than the past, when performing point matching.

Figure 18b shows the target path of the FV, $T_{F.FV}$, which is the final output of the proposed path planning algorithm. It can be observed that this target path is located outside of ${\bar{T}}_{R.FV}$ (or $T_{R.FV}$). This is an effort to overcome the off-tracking phenomenon which produces fairly large positive kingpin angle in both trucks in this case.

In Figure 18b, the LV truck was removed from the plot to demonstrate that the FV can generate a long-range target path even when its front view is severely impaired by the trailer of the LV. This is important since it can provide lateral string stability to the fleet. In fact, the conditions of Scenario S1 are too harsh to be found in actual driving situations.

#### 5.1.2. Scenario S2-Curved Road with 250R, 90 kph, 0.7 s Time Gap

**Figure 19:**
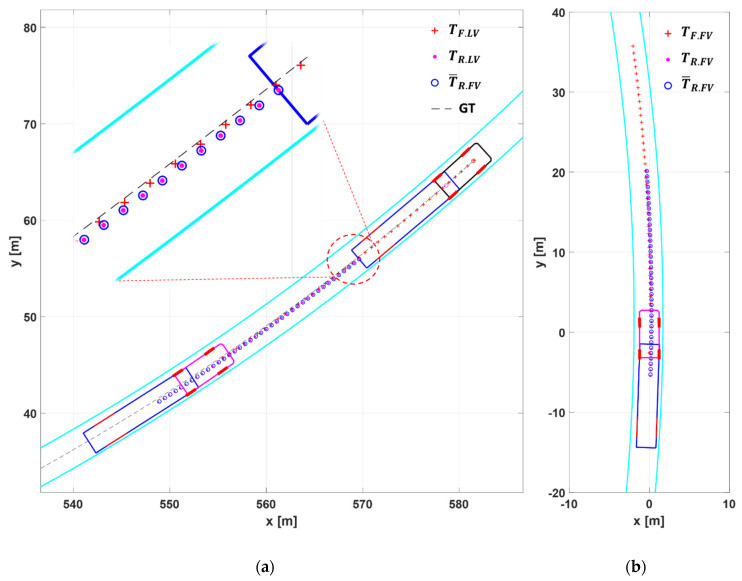
The simulation results for Scenario S2 (**a**) $T_{F.LV}$, $T_{R.LV}$, and ${\bar{T}}_{R.FV}$ in LV and FV; (**b**) $T_{F.FV}$, $T_{R.FV}$ and ${\bar{T}}_{R.FV}$ in FV.

Figure 19 shows the simulation results for Scenario S2.

Figure 19a shows that in the LV, the tractor trajectory $T_{F.LV}$ was formed outside the trailer trajectory $T_{R.LV}$, which was the opposite of what happened in Scenario S1. In Scenario S2, the lateral acceleration was 0.25 g—much higher than 0.13g in Scenario S1—and due to this large centrifugal force, the trailer was pushed outside to yield the high-speed off-tracking pattern in Figure 3b. Like in Scenario S1, the two trajectories ${\bar{T}}_{R.FV}$ and $T_{R.LV}$ matched almost exactly, which indicates the high reliability of the path planning algorithm of this paper. The deviation of $T_{F.LV}$ from its true trajectory (GT) can be explained similarly as in Scenario S1, except that at 90 kph, the look-ahead distance where the actual target path begins was about 35m ahead of the FV.

Figure 19b shows that the target path of the FV, $T_{F.FV}$, was located slightly inside of ${\bar{T}}_{R.FV}$ (or $T_{R.FV}$). With such configuration, the FV can prevent the vehicle from leaving its lane outward. This path must be very close to the actual trajectory of the LV truck, which was driven manually by an experienced driver.

For large cargo trucks, if the target path was incorrectly generated at high speeds, the lateral stability of the vehicle may be compromised, raising the risk of rollovers. Since the driver of LV is a professional driver who understands platoon driving well, the trajectory generated by LV driving at high speeds is the target path that can guarantee the stability of the vehicle. FV performs the proposed path planning using the trajectory of LV and directly follows the path, and then rollover can be prevented. In the same principle as feed-forward control using the target longitudinal acceleration of the LV for longitudinal control, using the LV trajectory received via V2V communication as a target path is a key to ensuring the string stability of the truck platoon.

#### 5.1.3. Scenario S3–DLC (Double Lane Change) on a Straight Road, 90 kph, 0.7 s Time Gap

**Figure 20:**
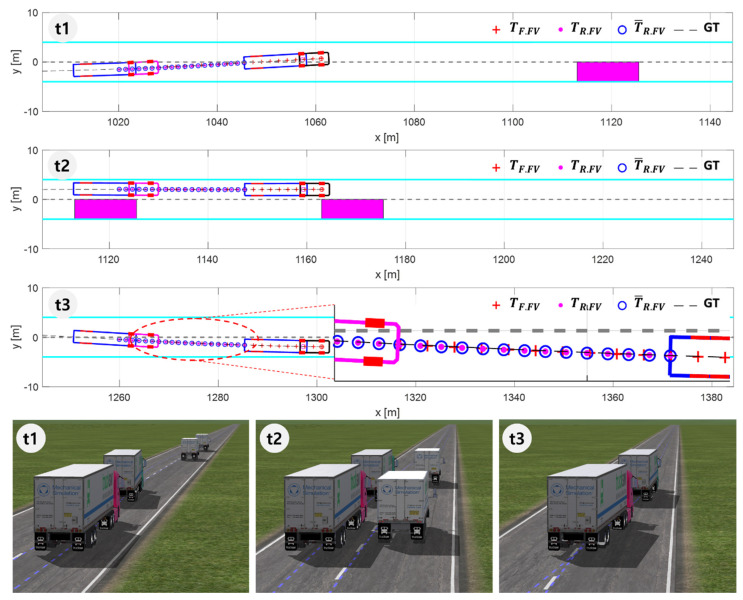
The simulation results for Scenario S3. At t1, LV finds stopping vehicles and begins to change lanes. At t2, the platoon is passing by them. At t3, the platoon has overtaken stopped vehicles and is returning to its original lane.

**Figure 21:**
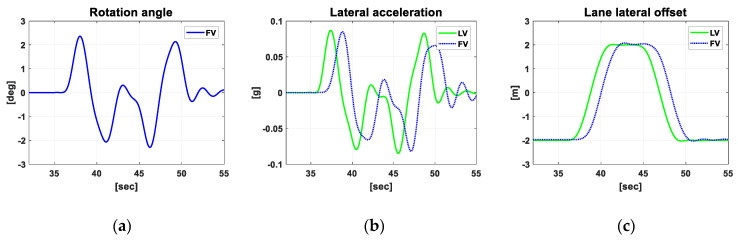
The simulation results for Scenario S3. (**a**) Rotation angle for point matching; (**b**) Lateral acceleration; (**c**) lateral offset of the vehicle with respect to the center of the lane.

Figure 20 and Figure 21 show the simulation results for Scenario S3 in which the platoon makes double lane change to avoid stopped vehicles ahead in their driving lane. While former scenarios were for verifying the steady-state performance of the proposed algorithm, this scenario is for verifying transient performance.

Figure 20 shows snapshots of the platoon at three different moments during DLC: t1 is when DLC initiates, t2 is when the platoon is passing the stopped vehicles, and t3 is when the platoon is returning to its original lane after securing enough space past the stopped vehicles.

Figure 20 shows that the FV recognizes the change in the driving path of the LV truck and successfully creates its target path throughout the DLC maneuver: During the transient periods of lane change and during the straight driving when passing the stopped vehicles.

Figure 21 shows that during the DLC the rotation angle computed in the point matching algorithm varies in the range of −2.3 deg to 2.4 deg, and the lateral acceleration varies between −0.085 g and 0.085g. This is rather a mild variation, whose amount depends on the platooning strategy for the lane change. As mentioned earlier, since the fleet length can reach 100 m in the platooning of articulated cargo trucks, the platoon can cause safety issues to the nearby vehicles. For this reason, lane change of the platoon must be minimized as much as possible, and even when it should happen as in the current scenario, both individual stability and string stability should not be violated. As shown in Figure 21b, the peak to peak of the lateral acceleration of the FV is slightly smaller than that of the LV. This means that the transient response characteristics have improved from the LV to the rear FV of the platoon, indicating that the lateral string stability has been secured. Figure 21c implies that the whole process of DLC is completed in 14 s, which corresponds to 350 m at 90 kph. Figure 21c also shows that the transient response of the platoon is quite stable, with almost negligible overshoot on both lane changes.

From the simulation results thus far, it could be verified that the proposed path planning algorithm provides lateral string stability for various driving conditions of truck platooning.

### 5.2. Road Test Experiments Result

**Figure 22:**
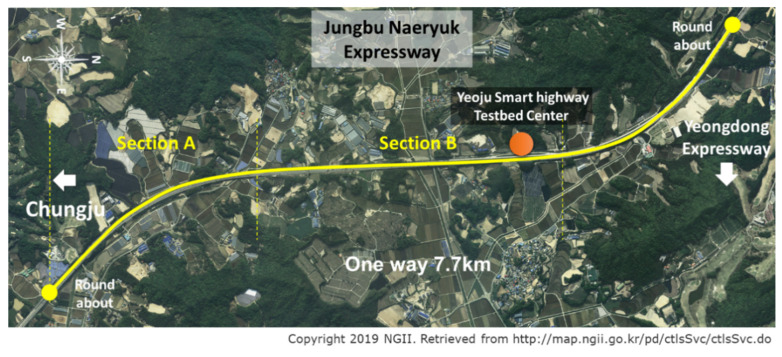
Yeoju Smart Highway testbed.

Along with simulation, actual vehicle tests were conducted to validate the proposed path planning algorithm. It was performed in the Yeoju Smart Highway, which was built by the Korean government in 2014 as a testbed for cooperative autonomous vehicles. It is located right next to the Jungbu Naeryuk Highway near Yeoju Junction and is a two-lane road with a total length of 7.7 km. Figure 22 shows the satellite photograph. It includes a straight road part and two 2000R curved road parts with opposite curvatures.

#### 5.2.1. Scenario T1–Curved Road with 2000R, 80 kph, 0.7 s Time Gap

**Figure 23:**
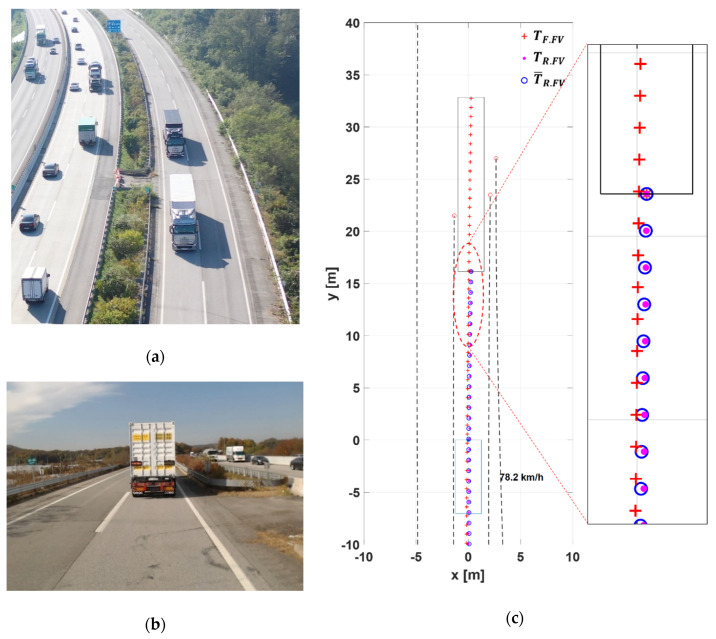
The test results for Scenario T1. (**a**) Top-view of the platooning trucks; (**b**) front-camera view of FV; (**c**) LV trajectories generated by FV in FV coordinate system.

Figure 23 shows the test results for Scenario T1. Using a drone, the image of the platooning trucks was recorded (Figure 23a), and the image of the LV’s rear view was captured by a camera installed in the FV (Figure 23b).

As can be imagined from the snapshots in Figure 23a,b, the platooning trucks could maintain the time gap without causing any lateral stability issues in this test. In Figure 23c, the front box represents the LV, and the rear box represents the FV’s tractor, and the black dotted lines on both sides of the vehicles indicate the road lanes that the camera perceived.

The plot in Figure 23c indicates that, with LV blocking the front view area of the FV’s camera, the lane detection range of the FV was not more 24 m ahead. Even worse, Figure 23b shows that the lane markings can be missing at some intervals in real situations. However, Figure 23c plot shows that despite these difficulties, the proposed path planning algorithm provides a reliable target path that spans 33 m ahead of the FV.

In Figure 23, it is very interesting to note that, although the road is gently curved to the right and the trucks are running at high speeds, the off-tracking is occurring in the opposite direction of the high-speed off-tracking pattern seen in Figure 3b. This is because the test road has a slight bank with a downside inside the curve, which is typical on a curved road of every highway. To a cargo truck, even a slight bank can notably affect its lateral motion by making the trailer slide down the bank by its own weight. Thus, the pattern of the target path in Figure 23c indicates that the proposed path planning algorithm is robust to environmental disturbances.

#### 5.2.2. Scenario T2–SLC (Single Lane Change) on a Straight Road, 80 kph, 0.7 s Time Gap

**Figure 24:**
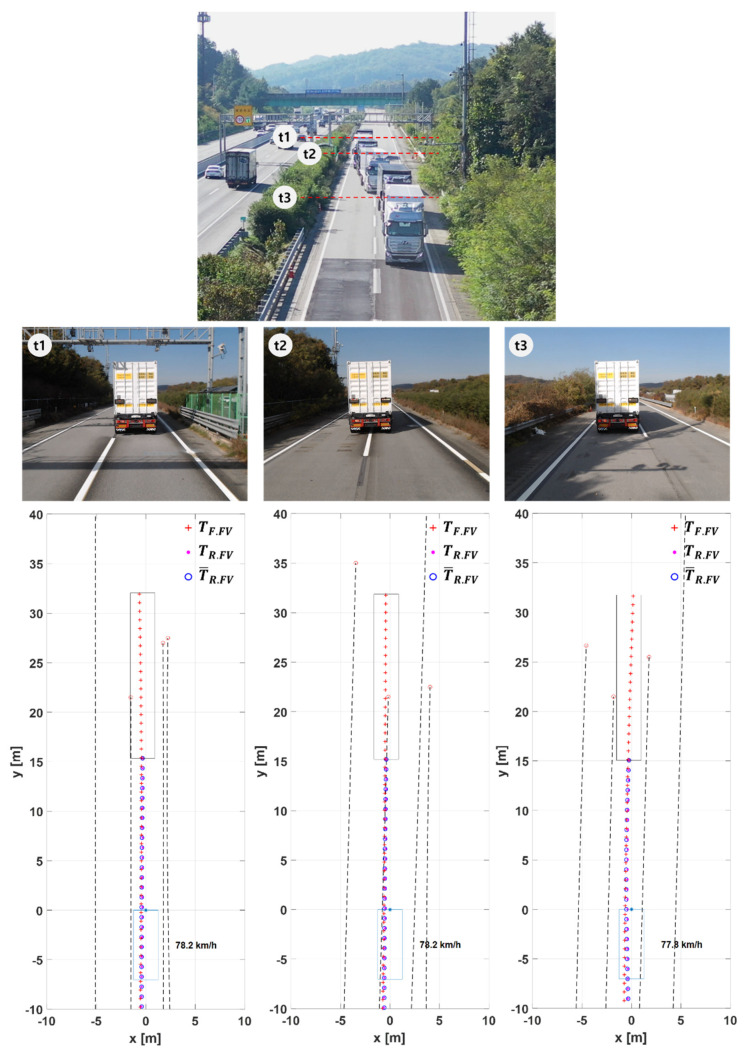
The test results for Scenario T2. At t1, LV initiates single lane change (SLC), at t2, FV changes lanes, and at t3, LV settles in the new lane.

Figure 24 shows the test results for Scenario T2. The first picture shows an overlapped image of three drone images shot at three different moments during SLC. The pictures on the second row show the snapshots of the LV at those three different moments.

In Figure 24, t1 is the time when the LV initiates SLC to the left lane. The bottom graphs show that at t1 the target path of the FV is properly generated toward the left lane. If the FV was relying only on camera/radar to generate its target path, it would have been hard to tell whether the LV intended to change lanes or else (like when the trailer of the LV unintentionally drifts out of its lane due to a bank). Using V2V communication and the proposed algorithms, however, the path planning method could tell the LV’s intention and generate the target path of the FV to agree with the intention.

From t2 to t3, the FV changes lanes and then settles on the new lane. Similarly to the above case, if the FV follows the rear point of the LV that its front camera/radar perceives, the FV would experience significant overshoot to settle on the new lane by knowing the LV’s intention only after its result occurs. In a typical single lane change maneuver, an experienced driver is known to perform reverse steering as early as when the vehicle is in the middle of crossing lanes to reduce the overshoot later when the vehicle settles on the new lane. In this test, it was observed that the proposed path planning algorithm worked just like an experienced driver with enough look-ahead distance virtually made.

As shown in the front camera view in the middle of Figure 24, a downward slope is formed in the direction of changing lanes. Heavy-duty trucks are at risk of being pushed down the slope by the load, and the risk will be increased when changing lanes. The FV needs to quickly control the vehicle’s attitude through reverse steering at the appropriate point during the lane change. The proposed algorithm enables the FV to immediately respond to the steering intention that the professional LV driver responds to changes in the road, thus that the FV can stably follow the LV in any road environment.

From the results by simulation and experimental tests, the path planning algorithm of this paper has demonstrated capabilities to respond quickly to the LV’s steering intention and to act against unexpected road disturbances, which can enable more sophisticated steering control and thus secure lateral string stability of the platoon.

#### 5.2.3. Scenario T3–Unintended Steering Input, 80 kph, 0.7 s Time Gap

**Figure 25:**
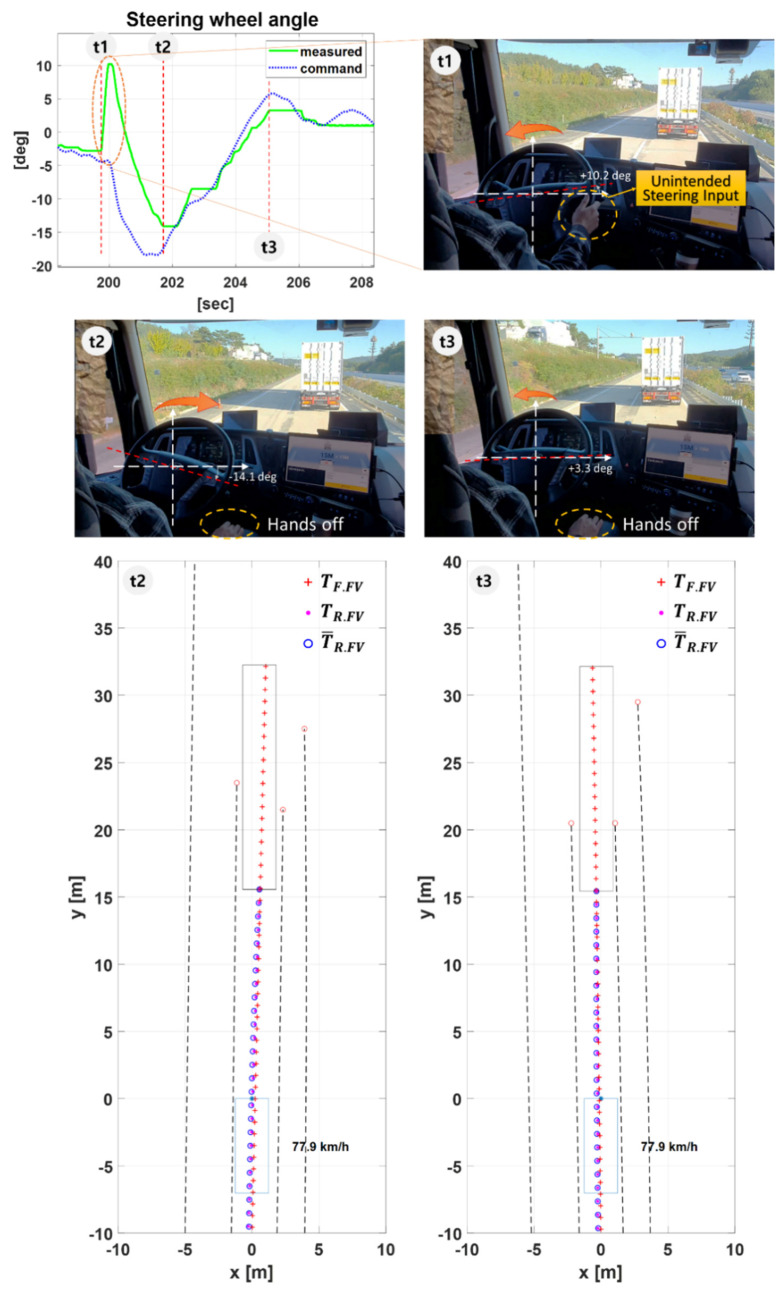
The test results for Scenario T3. At t1, FV’s driver make a steering input, at t2, FV is returning to the center of the lane, and at t3, FV settles in the lane.

Figure 25 shows the test results for Scenario T3. This scenario was considered to validate the reliability of the proposed path planning algorithm in a situation where an unintended steering disturbance is applied to the vehicle. The first graph shows the measured steering wheel angle and steering wheel angle command. The pure pursuit algorithm [26] was used for path tracking. Three photos of the inside of the tractor’s cab are shown in chronological order from t1 to t3.

At t1, the FV driver turned the steering wheel counterclockwise to make an unintended steering disturbance into the vehicle. As shown in the first graph in Figure 25, a steering wheel angle of 10.2 deg was applied to FV. The driver released his hands from the steering wheel immediately after making the steering input. At t2, while moving out of the lane, the FV generates the target path that the LV, which is running normally, traveled based on the coordinate system of the FV. At this time, steering wheel angle command was generated up to −18 deg to follow the trajectory of the LV. At t3, FV is stably converging through a slight opposite steering after reaching the target path. Through scenario T3, it was verified that the FV can follow the LV by stably generating the target path even when steering disturbance occurs.

In road test scenarios T1, T2, and T3, the proposed path planning algorithm is robust and functioning accurately in various driving environments. By receiving the trajectories of the LV via V2V communication, it is possible to effectively solve the problem of the limited perceived distance of the existing path tracking algorithm for platooning. First, it is possible to overcome an error between the LV’s tractor trajectory and the FV’s trajectory by following the trajectory of the tractor rather than the trailer in consideration of the off-tracking characteristics of the truck. Next, by using the path reflecting the steering intention of the professional driver of the LV, the FV can generate a target path that is robust against changes in road conditions such as banks. Lastly, it was possible to create a target path considering the influence of the driver’s unintended steering input as well as the external disturbance factor.

## 6. Conclusions

Truck platooning refers to a form in which a number of trucks run as a fleet with short inter-vehicle distance using V2V communication. The leading vehicle is driven manually by an experienced driver, and the following vehicles run by autonomous driving. To successfully perform platooning in various situations, each truck must maintain dynamic stability and at the same time, the whole system must maintain string stability.

Due to the short front-view range, the following vehicles’ path planning capabilities become significantly impaired. In addition, in platooning with articulated cargo trucks, which is the case of this study, an off-tracking phenomenon occurring on a curved road makes it hard for the following vehicle to track the trajectory of the preceding truck. Furthermore, without knowledge of the global coordinate system, it is difficult to correlate the local coordinate systems that each truck relies on for sensing environment and dynamic signals.

In this paper, to solve these problems, a path planning algorithm for platooning of articulated cargo trucks has been developed. Using the Kalman filter, V2V communication, and a novel update-and-conversion method, each following vehicle can accurately compute the trajectory of the leading vehicle’s front part for using it as a target path. This paper’s path planning algorithm was validated by simulations on severe driving scenarios and by tests on an actual road. From the simulation and experimental results, it could be verified that the proposed path planning algorithm provides lateral string stability, even for very harsh driving conditions of truck platooning. The algorithm also demonstrated the capabilities to respond quickly to the leading vehicle’s steering intention and to act against unexpected road disturbances, which can enable sophisticated path tracking control.

## Figures and Tables

**Table 1:** The message that the leading vehicle (LV) sends to the following vehicle (FV) via V2V communication.

Message	Notation
coefficients of 3rd order polynomial for LV’s “Front” trajectory	$\left[C_{F_{3}},\;C_{F_{2}},\;C_{F_{1}},\;C_{F_{0}}\right]$
coefficients of 3rd order polynomial for LV’s “Rear” Trajectory	$\left[C_{R_{3}},\;C_{R_{2}},\;C_{R_{1}},\;C_{R_{0}}\right]$
coordinate of LV’s “Rear” point at the current sample	$\left[x_{R.LV_{1}},\;y_{R.LV_{1}}\right]$

**Table 2:** Trajectories involved in path planning.

Mathematical Notation	Description
$T_{F.LV}=\left[\begin{array}{c} X_{F.LV}\\ Y_{F.LV} \end{array}\right]=\left[\begin{array}{c} x_{F.LV_{1}}\;x_{F.LV_{2}}\;\cdots \;x_{F.LV_{n}}\\ y_{F.LV_{1}}\;y_{F.LV_{2}}\;\cdots \;y_{F.LV_{n}} \end{array}\right]$	trajectory of “Front” point in LV’s coordinate system
$T_{R.LV}=\left[\begin{array}{c} X_{R,LV}\\ Y_{R,LV} \end{array}\right]=\left[\begin{array}{c} x_{R.LV_{1}}\;x_{R.LV_{2}}\;\cdots \;x_{R.LV_{n}}\\ y_{R.LV_{1}}\;y_{R.LV_{2}}\;\cdots \;y_{R.LV_{n}} \end{array}\right]$	trajectory of “Rear” point in LV’s coordinate system
$T_{F.FV}=\left[\begin{array}{c} X_{F.FV}\\ Y_{F.FV} \end{array}\right]=\left[\begin{array}{c} x_{F.FV_{1}}\;x_{F.FV_{2}}\;\cdots \;x_{F.FV_{n}}\\ y_{F.FV_{1}}\;y_{F.FV_{2}}\;\cdots \;y_{F.FV_{n}} \end{array}\right]$	trajectory of “Front” point in FV’s coordinate system
$T_{R.FV}=\left[\begin{array}{c} X_{R,FV}\\ Y_{R,FV} \end{array}\right]=\left[\begin{array}{c} x_{R.FV_{1}}\;x_{R.FV_{2}}\;\cdots \;x_{R.FV_{n}}\\ y_{R.FV_{1}}\;y_{R.FV_{2}}\;\cdots \;y_{R.FV_{n}} \end{array}\right]$	trajectory of “Rear” point in FV’s coordinate system
${\bar{T}}_{R.FV}=\left[\begin{array}{c} {\bar{X}}_{R,FV}\\ {\bar{Y}}_{R,FV} \end{array}\right]=\left[\begin{array}{c} {\bar{x}}_{R.FV_{1}}\;{\bar{x}}_{R.FV_{2}}\;\cdots \;{\bar{x}}_{R.FV_{n}}\\ {\bar{y}}_{R.FV_{1}}\;{\bar{y}}_{R.FV_{2}}\;\cdots \;{\bar{y}}_{R.FV_{n}} \end{array}\right]$	trajectory of “Rear” point generated by FV

**Table 3:** Test scenarios for simulation.

No.	Speed	Time-Gap	Method	Radius [m]
Scenario S1	40 kph ^1^	0.7 s ^1^	Driving on a curved road	100R
Scenario S2	90 kph ^1^	0.7 s	Driving on a curve road	250R
Scenario S3	90 kph	0.7 s	Double lane change	Straight road

^1^ In the TROOP project, the minimum speed of platooning is 40 kph, the maximum speed is 90 kph, and the target time-gap for demonstration of platooning in 2020 is 0.7 s.
